# Malaria policies *versus* practices, a reality check from Kinshasa, the capital of the Democratic Republic of Congo

**DOI:** 10.1186/s12889-015-1670-0

**Published:** 2015-04-10

**Authors:** Hypolite Muhindo Mavoko, Gillon Ilombe, Raquel Inocêncio da Luz, Albert Kutekemeni, Jean-Pierre Van geertruyden, Pascal Lutumba

**Affiliations:** Département de Médecine Tropicale, Université de Kinshasa, B.P. 747, Kin XI, Kinshasa, République Démocratique du Congo; International Health Unit, Department of Epidemiology, University of Antwerp, Campus Drie Eiken, Universiteitsplein 1, 2610 Kinshasa, République Démocratique du Congo; Programme National de Lutte contre le Paludisme, Ministère de la Santé Publique, Kinshasa, République Démocratique du Congo

**Keywords:** Malaria, Policies, Practices, Health seeking behaviour, Phytomedicines, Democratic Republic of Congo

## Abstract

**Background:**

Artemisinin-based combination therapy (ACT) following a confirmed parasitological diagnosis is recommended by the World Health Organization (WHO) and the Congolese National Malaria Control Program (NMCP). However, commitment and competence of all stakeholders (patients, medical professionals, governments and funders) is required to achieve effective case management and secure the “useful therapeutic life” of the recommended drugs. The health seeking behaviour of patients and health care professionals’ practices for malaria management were assessed.

**Methods:**

This was an observational study embedded in a two-stage cluster randomized survey conducted in one health centre (HC) in each of the 12 selected health zones in Kinshasa city. All patients with clinical malaria diagnosis were eligible. Their health seeking behaviour was recorded on a specific questionnaire, as well as the health care practitioners’ practices. The last were not aware that their practices would be assessed.

**Results:**

Six hundred and twenty four patients were assessed, of whom 136 (21.8%) were under five years. Three hundred and thirty five (55%) had taken medication prior to the current consultation (self -medication with any product or visiting another HC) of whom 47(14%) took an antimalarial drug, and 56 (9%) were treated presumptively. Among those, 53.6% received monotherapy either with quinine, artesunate, phytomedicines, sulfadoxine-pyrimethamine or amodiaquine. On the other side, when clinicians were informed about laboratory results, monotherapy was prescribed in 39.9% of the confirmed malaria cases. Only 285 patients (45.7%) were managed in line with WHO and NMCP guidelines, of whom 120 (19.2%) were prescribed an ACT after positive blood smear and 165 (26.4%) received no antimalarial after a negative result.

**Conclusion:**

This study shows the discrepancy between malaria policies and the reality on the field in Kinshasa, regarding patients’ health seeking behaviour and health professionals’ practices. Consequently, the poor compliance to the policies may contribute to the genesis and spread of antimalarial drug resistance and also have a negative impact on the burden of the disease.

## Background

Approximately half of the global population is exposed to malaria [1]. Early parasitological diagnosis and efficacious treatment in all age groups are recommended by the World Health Organization (WHO) as key strategies to control malaria [2]. However, next to policies, availability of good quality drugs and parasite based diagnostic is essential [3]. Other important factors are access to health care services as well as the health seeking behaviour of the population. Policies are principally based on high quality research evidence, but implementation remains challenging on various levels because of some field realities.

In Sub Saharan Africa, which holds the gross of the malaria burden, the proportion of people treated for malaria with a confirmed diagnosis is low compared to other regions of the world [4]. In addition, the accuracy of malaria diagnosis at primary health care has been reported to be poor in many areas [5-8]. This may explain the fact that health practitioners tend to prescribe antimalarial drugs regardless of laboratory results. Furthermore, patients can purchase antimalarial drugs directly on the informal market without any medical prescription [9,10].

In 2010, only a few months after its publication, the Congolese National Malaria Control Program (NMCP) adopted the second edition of the WHO malaria treatment guidelines [2]. The major novelty was the recommendation to give antimalarial treatment upon parasitological evidence of malaria infection even in children under the age of five years. The purpose was to improve targeting of treatment and better quality of care, in particular in an environment of increased malaria control and or declining malaria endemicity [2]. Indeed non malaria cases would be identified and may benefit from timely treatment thereby reducing morbidity and mortality. In DRC, confirmed uncomplicated malaria cases are recommended to be treated either with artesunate+amodiaquine (ASAQ) or artemether+lumefantrine (AL) as the first line, and quinine+antibiotic having antimalarial activity as second line [11].

Improving malaria case management requires the involvement of all stakeholders and detection of the operational bottle necks. Inspired by the Piot Model [12], the health seeking behaviour of the patients and practices of the health care providers were assessed going from the first presumed malaria symptoms until the delivery of care in formal health centres (HC). The objective was to assess health seeking behaviour of patients and practices related to the management of uncomplicated malaria by the health professional at primary health care level (PHC) compared to the policies.

## Methods

### Study setting and design

This was an observational study embedded in a two-stage cluster randomized survey conducted in Kinshasa, the capital city of the Democratic Republic of Congo (DRC). This city is divided into 35 health zones among them 12 were randomly selected: Bandalungwa, Binza ozone, Bumbu, Kimbanseke, Kingabwa, Kokolo, Limete, Makala, Maluku 1, Masina 1, Police and Selembao. In each health zone, one public or private HC was randomly selected among those reporting to the National Health Information System. The former study consisted of evaluating the accuracy of Optimal-IT®, as well as Paracheck-Pf® malaria rapid diagnostic tests (RDTs) [5]. RDTs were performed and microscopy slides prepared at recruitment. HC laboratory technicians had to read the slides and were blinded to the RDTs results. The same slides were read by expert microscopists for quality control (QC) and they were blinded to previous results. The QC results constituted the gold standard to assess the accuracy of RDTs and microscopy at PHC level. The sample size of 624 was calculated for the main study based on the accuracy of Optimal-IT® RDT and the same number was involved in these supplementary analyses.

Data were collected from May to June 2011. All the patients attending the health facility and clinically diagnosed with malaria and to whom a blood smear (BS) was requested, were eligible. Clinical diagnosis was established by clinicians based on unspecific known malaria symptoms like fever, history of fever, headache, chills, weakness, muscle and joint pain and anorexia. Data was collected in 2 phases. First, a questionnaire was filled during an interview. Socio-demographic data were recorded and the patient/guardians’ health seeking behaviour was assessed i.e. current illness, action taken and health seeking practices prior to the current visit. In a second phase, the medications (antimalarial and others) prescribed by the clinicians before and after the BS results as well as the laboratory results were also recorded. Clinicians were not informed on the data collection to avoid observer’s bias. Antimalarial drugs taken before seeking care and/or prescribed at the PHC were identified using the list of antimalarial drugs available on the market in Kinshasa (e.g. Artemether+lumefantrine: Coartem®, Coartesiane®, Lonart®, Luther®, etc.).

### Laboratory examination

The collected BSs were stained with 10% Giemsa for 10 minutes. Thin BSs were fixed with methanol prior to the staining. Microscopy reading was first performed by the HC laboratory technicians and result was given to clinicians, who were supposed to manage the cases according to parasitological diagnosis and National guidelines and policies [11]. Slides were subsequently transported to the parasitology unit, Kinshasa University for QC.

### Data analysis

Data were double-entered and validated in Epi info version 3.5.1 software and analysed using Stata version 11 (Stata Corp, Lakeway, College Station, Texas, USA). The primary outcome was the proportion of patients to whom a recommended antimalarial drug was prescribed after a positive test and those for whom no prescription was made after a negative test. Secondary outcomes included description of patients’ health seeking behaviour (time for consultation regarding the illness episode onset, medication taken before consultation) and malaria testing outcome compared to patients’ health seeking behaviour and clinicians’ practices. Descriptive statistics were used to get frequencies and percentages. Cross tabulations were performed to check relationship between variables of interest using the Chi-square test of Pearson. Predictors of malaria confirmation were assessed by a logistic regression, referring to experts’ microscopy reading result. The cluster effect was checked and did not influence the results.

### Ethical approval

The study was approved by the Committee for Medical Ethics of the Antwerp University Hospital, Belgium (approval reference: 14/36/236) and the Ethical Committee of the School of Public Health, Kinshasa University, DRC (approval reference: ESP/CE/082/10). Before inclusion, written informed consent was obtained from all patients or their legal guardians if minors.

## Results

### Baseline characteristics

A minimum of 50 patients were enrolled from each HC, with a total of 624. Of those, 136 were under five years (21.8%). In general, history of fever was the most frequent symptom (59.8%), and it was significantly more reported in children under five years compared to other age groups (p < 0.001) (Table 1). At PHC, 361 patients (57.9%) tested positive for malaria, whereas only 123 (19.7%) were confirmed by the experts’ reading.

**Table 1:** **Baseline characteristics of 624 patients in primary health care setting, Kinshasa, DRC, 2011**

**Variable**	**All age group**	**<5 years**	**≥5 year**	**p**
	**(N = 624)**	**(N = 136)**	**(N = 488)**	
	**n (%)**	**n (%)**	**n (%)**	
**Female (n, %)**	362 (58.0)	62 (45.6)	300 (61.5)	0.001
**History of fever**	373 (59.8)	123 (90.4)	250 (51.2)	<0.001
**Ownership mosquito net**	333 (53.4)	76 (55.8)	257 (52.7)	0.51
**Slept under mosquito net last night**	224 (35.9)	56 (41.1)	168 (34.4)	0.15
**Malaria self-treatment prior current consultation using any antimalarial drug**	100 (16.0)*	18 (13.2)	82 (16.8)**	0.31

*out of 623; **out of 487.

### Care seeking practice

**Figure 1:**
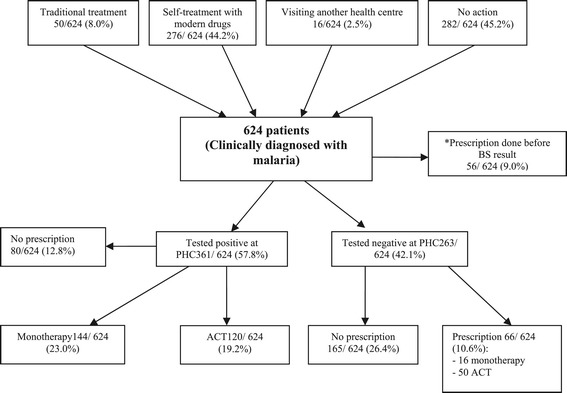
**Flow chart of the patients’ and clinicians’ practices in primary health care setting, Kinshasa, DRC, 2011.**

Among 373 patients who reported history of fever, 24 (6.4%) attended the HC the same day when fever started, 55 (14.8%) the following day, 154 (41.3%) after 2–3 days, 95 (25.5%) after 4–7 days, whereas 45 (10.1%) waited for more than a week. Lowest proportion of malaria was confirmed (both at PHC and by experts) among those who came more than a week after the fever onset (Table 2). At the same time, malaria was less confirmed in the group that attended the HC directly (Table 2). Before the current consultation, 50 patients (8%) had taken traditional treatment, 276 (44.2%) resorted to self-treatment with modern drugs, 16 (2.8%) visited another HC previously. Two hundred and eighty two (45.2%) did not undertake any action prior to the current visit (Figure 1). Before attending the HC, 100 patients (16%) had taken an antimalarial drug, 288 (46.2%) took another type of drug (antibiotics, antipyretic, etc.), and 235 (37.8%) took no drugs. Patients who had taken no antimalarial drugs more likely attended the HC during the first 3 days of fever onset (p = 0.02).

**Table 2:** **Diagnosis outcome compared to the patients’ health seeking practice in primary health care setting, Kinshasa, DRC, 2011**

**Patients’ practice**	**Malaria cases confirmed at PHC**	**Malaria cases confirmed by experts**
	**n (%)**	**n (%)**
**Days from fever onset to the current consultation (n/ N)**		
**0 (24/ 373)**	17 (70.8)	7 (29.2)
**1 (55/ 373)**	30 (54.6)	15 (27.3)
**2 to 3 (154/ 373)**	102 (66.2)	50 (32.5)
**4 to 7 (95/ 373)**	61 (64.2)	21 (22.1)
**>7 (45/ 373)**	26 (57.8)	9 (20.0)
**Care seeking bahaviour**		
**Self-treatment with traditional products (50/ 624)**	33 (66.0)	12 (24.0)
**Self-treatment with modern products (276/ 624)**	166 (60.1)	72 (26.1)
**Visited another HC (16/ 624)**	9 (56.3)	2 (12.5)
**No action (282/ 624)**	153 (54.3)	37 (13.1)

### Prescribing practice

Only 285 cases (45.7%) were managed in line with WHO guidelines as ACTs were prescribed to 120 positive cases and nothing to 165 negative cases (Figure 1). ACTs consisted of ASAQ (100 cases, 30.0%), AL (63 cases, 19.0%) and artesunate+sulfadoxine-pyrimethamine (AS+SP) (7 cases, 2.1%). Monotherapy consisted of quinine (88 cases, 26.7%), artesunate (31 cases, 9.4%), phytomedicines (24 cases, 7.3%), sulfadoxine-pyrimethamine (16 cases, 4.8%) and amodiaquine (1 case, 0.3%).

Fifty six patients (9.0%) received an antimalarial prescription while BS results were still unknown and this was observed more frequently in children under five years, but the difference was not significant (p = 0.54). Quinine (34.0%), AL (34.0%) and SP (10.7%) were the most prescribed antimalarials in these cases. Clinicians changed the prescription for 7 of them in light of the BS results from SP to ACT. Prior treatment before attending the HC did not influence prescription behavior (p = 0.09).

For 330 patients (53.0%), antimalarial prescription was done based on BS results. In that case, ASAQ was most frequently prescribed (30.3%), followed by quinine monotherapy (26.7%) and AL (19.1%). Monotherapy was prescribed to 39.8% of patients who tested positive for malaria at PHC. Malaria was more likely confirmed by experts’ reading when the patients had reported history of fever (p < 0.001), undertook no treatment before attending the HC (p < 0.001) and lacked mosquitonet (p = 0.05). In the multivariate analysis, history of fever, treatment seeking behaviour and not sleeping under mosquitonet were significantly associated with malaria confirmation (Table 3).

**Table 3:** **Malaria confirmed by experts’ reading based on practices of patients and medicals in primary health care setting, Kinshasa, DRC, 2011**

**Variable (n)**	Blood smear positive (%)*	**OR (95% CI)**	**p**	Adj OR* (95% CI)	**p**
**Age group**					
**<5 years (136)**	33 (24.3)	1.4 (0.9-2.3)	0.13	-	NS
**≥5 years (488)**	90 (18.4)	1			
**History of fever**					
**Yes (373)**	102 (27.4)	4.1 (2.5-7.1)	<0.001	3.6 (2.1-6.1)	<0.001
**No (251)**	21 (8.4)	1		1	
**Antimalarial treatment prior current consultation**					
**Yes (100)**	17 (17)	1		-	
**No (523)**	106 (20)	0.8 (0.4-1.4)	0.45		NS
**Treatment seeking practice**					
**Yes (342)**	86 (25.2)	2.2 (1.4-3.5)	<0.001	1.6 (1.0-2.5)	0.04
**No (282)**	37 (13.1)	1			
**Mosquitonet ownership**					
**Yes (333)**	56 (16.8)	1	0.05	-	NS
**No (291)**	67 (23.0)	1.4 (1.0-2.4)			
**Slept under mosquito net last night**					
**Yes (224)**	35 (15.6)	1	0.40	1	0.02
**No (109)**	21 (19.3)	1.3 (0.7-2.4)		1.7 (1.0-2.6)	
**Malaria treatment prescribed before blood smear result**					
**Yes (56)**	16 (28.6)	0.6 (0.3-1.2)	0.08	-	NS
**No (568)**	107 (18.9)	1			

*Malaria confirmed by experts.

## Discussion

Results of this survey highlight the alarming situation of malaria management at PHC in the DRC because of a huge discrepancy between policy and field reality. Almost half of the patients (45.7%) were managed in line with NMCP guidelines. However, 7 patients (2.1%) received a prescription for AS+SP which is not officially recommended in DRC but still in line with the WHO recommendations. Besides, a proper prescription is not enough to ensure accurate malaria management. The steps forward are availability of quality-assured antimalarial drugs and the patient/ care giver’s compliance to the treatment [13,14]. Nevertheless, this study did not explore those aspects. The lack of compliance with NMCP guidelines has been reported elsewhere in sub-Saharan Africa [15-20]. Malaria control programs need to elaborate an efficient strategy for the follow up of adherence to guidelines by practitioners. In addition, a dialogue between policy makers, researchers and program managers is needed to discuss and address the gaps in the implementation of malaria management policies [21].

The overtreatment reported in this survey could be the result of unsatisfactory diagnostic accuracy at PHC [5,22]. This may explain why clinicians seldom relied on the BS results. Another explanation could be related to the mistrust of policies evidence, as reported in other studies [23,24]. In Tanzania, Reyburn *et al.* [25] found that almost half of patients with a negative malaria test were treated with an antimalarial drug. The responsibility of the overtreatment is to be shared with the patients who can influence health practitioners’ practice [26,27]. In Ghana, participants to a focus group discussion claimed that a test should result in a diagnosis, even following a negative result [28]. In this aspect, clinicians may choose to prescribe antimalarial drugs, mostly when laboratory diagnoses for other infections are not available.

Clinicians tended to prescribe more antimalarial drugs to children younger than five, even when laboratory results were negative. Clear guidelines for management of non-malaria febrile illnesses as well as appropriate tests and drugs are urgently needed [29]. By the time this survey was conducted, ASAQ and AL were the recommended first line ACTs. It was noticed that clinicians tended to prescribe more ASAQ when BS result was positive. When prescription was done before BS or in spite of negative BS result, either Quinine or AL was preferred. Assuming that they were not aware about the introduction of AL in the national guidelines some months prior to this assessment, the hypothesis is that they had preference for non-recommended antimalarial drugs in absence of malaria confirmation.

Combination treatment using ACTs is the key strategy to reduce the impact of failing monotherapies [30,31]. Unfortunately and against the national guidelines, this survey highlighted a worrying proportion of prescribing monotherapy, including artemisinin monotherapy (AMT), a practice that could increase treatment failures and contribute to the spread of resistance. Despite the WHO’s call to stop AMT production and marketing to refrain the development of drug resistance [31], the field reality is still different. However, a trend towards their disappearance from the market in Kinshasa is notably observed. In 2009 they constituted 10% and 41% of antimalarial drugs stocked in public/ Not-for-Profit and private sector outlets respectively [13]. Four years later their availability decreased below 1% in both sectors [32].

It is worth mentioning the ongoing initiative in the country supported by the Global Fund that aims to supply RDTs as well as recommended medications. The support is presently available in 60% of the health zones in DRC. At the time this survey was conducted, two of the twelve HC involved were supplied, although on an irregular basis.

Apparently, none of the clinicians involved was aware, 6 months after the NMCP guidelines update, that quinine should not be used as a monotherapy. This survey did not focus on the knowledge nor the background of practitioners and further research will be needed to assess explanatory causes and identify the bottlenecks where training or adapted measurements can be taken. The lack of this information is a limitation of the study, but these findings may be reproducible in other settings.

Almost half of the patients (44.2%) undertook a treatment prior to the current consultation and they tended to be more infected with malaria. This means that either the medication taken was not effective or the treatment course was not appropriate. Most of them used antimalarial, antibiotics and antipyretics. Such inappropriate use of antibiotics and antimalarials is dangerous as it may contribute to the emergence and spread of drug resistant bacteria and *Plasmodium* [29,33]. Self-treatment has been reported to be common, mainly in rural areas of Africa [9]. People should be encouraged to address to the health facilities as soon as the clinical episode starts, for proper diagnosis and treatment. In the urban settings like Kinshasa, geographical access is not the main obstacle but affordability of the health services can be the main limiting factor. This could explain why few people with history of fever attended the HC the same day. Access to health may be increased by increasing the health insurance coverage either through the Government or the private stakeholders.

Approximately a third (35.9%) of those owning a mosquitonet slept under it the night before consultation. The protective impact on sleeping under mosquitonet was shown by the multivariate analysis. Hence the campains are highly needed to sensitize the mass on the benefits of using the mosquitonet.

## Conclusion

Data of this study indicate that neither the health seeking behaviour of the patients nor the treatment practices of the health care providers were appropriate. This may have a negative impact on the burden of the disease and contribute to the genesis and spread of drug resistance. However, national malaria related health policies are in line with WHO recommendations and regularly updated. Health education, including health promotion and training on fever management is urgently needed.
